# Occupational Exposure to Talc Increases the Risk of Lung Cancer: A Meta-Analysis of Occupational Cohort Studies

**DOI:** 10.1155/2017/1270608

**Published:** 2017-08-31

**Authors:** Che-Jui Chang, Yu-Kang Tu, Pau-Chung Chen, Hsiao-Yu Yang

**Affiliations:** ^1^Institute of Occupational Medicine and Industrial Hygiene, National Taiwan University College of Public Health, Taipei, Taiwan; ^2^Department of Family Medicine, National Taiwan University Hospital, Taipei, Taiwan; ^3^Department of Public Health, National Taiwan University College of Public Health, Taipei, Taiwan; ^4^Institute of Epidemiology and Preventive Medicine, College of Public Health, National Taiwan University, Taipei, Taiwan; ^5^Department of Environmental and Occupational Medicine, National Taiwan University Hospital, Taipei, Taiwan; ^6^Department of Environmental and Occupational Medicine, National Taiwan University College of Medicine, Taipei, Taiwan

## Abstract

**Objective:**

Talc is widely used in industrial applications. Previous meta-analyses of carcinogenic effects associated with inhaled talc included publications before 2004, with a lack of data in China, the largest talc-producing country. The safety of workers exposed to talc was unclear due to limited evidence. The objective of this study was to reevaluate the association between inhaled talc and lung cancer.

**Setting, Participants, and Outcome Measures:**

A meta-analysis was performed to calculate the meta-SMR of lung cancer. We searched the MEDLINE, EMBASE, CNKI, and Wanfang Data databases through March 2017. Data from observational studies were pooled using meta-analysis with random effects models.

**Results:**

Fourteen observational cohort studies (13 publications) were located via literature search. The heterogeneity of the included data was high (*I*-squared = 72.9%). Pooling all the cohorts yielded a meta-SMR of 1.45 (95% CI: 1.22–1.72, *p* < 0.0001) for lung cancer among the study subjects exposed to talc. Subgroup analysis for asbestos contamination showed no significant difference in lung cancer death between subjects exposed to talc with and without asbestos (*p* = 0.8680), indicating that this confounding factor may have no significance.

**Conclusions:**

This study provides evidence that nonasbestiform talc might still increase the risk of lung cancer. Further epidemiological studies are required to evaluate the safety of workers with occupational talc exposure.

## 1. Introduction

Talc is a mineral that is commonly used in food, drug, and cosmetic and industrial applications. Due to the platyness, softness, hydrophobicity, organophilicity, and inertness of talc, it can bring benefits to a wide range of industries, including agriculture, ceramics, food, paper, pharmaceuticals, plastics, and rubber [1].

Regarding the carcinogenicity of talc, we must distinguish between talc with and without asbestos. In a review published by the International Agency for Research on Cancer (IARC) in 1987, talc powders containing and not containing asbestiform fibers were separated as topics of discussion [2]. The IARC classifies talc containing asbestos as “carcinogenic to humans” (group 1). However, based on limited data of animal studies and lack of evidence in human studies, the IARC classifies inhaled talc without asbestos as “not classifiable as to carcinogenicity in humans” (group 3) [3]. Although coexposure of talc and asbestos is common, talc was much less discussed and regulated. For instance, the U.S. National Toxicology Program (NTP) has not fully reviewed talc as a possible carcinogen [4].

It is worth noting that major reviews of human studies of talc are largely based on epidemiologic studies conducted in Europe and North America. Talc production occurs worldwide, and China is the leading producer of talc in the world, followed by India, Brazil, and the United States. China produced 2,200 thousand metric tons of talc in 2013, more than three times that of the second largest producer, India, which produced 663 thousand metric tons of talc in the same year [5]. However, systematic reviews or meta-analyses have seldom cited the literature related to talc exposure and epidemiological studies from China. This may be because studies of talc in China are usually written in Chinese, with only their titles translated into English. Therefore, in the present study, we included Chinese studies in the meta-analysis to obtain more comprehensive results.

The objective of this study was to evaluate how occupational inhaled talc exposure affects lung cancer risk.

## 2. Methods

We conducted a meta-analysis by pooling data from eligible occupational cohorts exposed to talc.

### 2.1. Literature Search

A study protocol was prospectively developed according to the MOOSE guidelines [19]. A literature search was performed in the online reference databases MEDLINE and EMBASE for papers published worldwide. The last update of the literature search was in March 2017. We also searched the China National Knowledge Infrastructure (CNKI) and Wanfang Data, two major Chinese bibliographic databases, because many studies on occupational talc exposure have been published in Chinese journals. The following search terms were used: “talc and cancer” and “talc and mortality”. For example, the search query for MEDLINE was “(talc AND mortality) OR (talc AND cancer)”. We translated the above query terms into Chinese to search CNKI and Wanfang Data. The results of the literature search were stored on Endnote X7 for the detection of duplicates [20]. For cohorts that were investigated in more than one eligible study, all data were retrieved from the most recent article. The bibliographies of the identified papers were then examined for additional relevant articles, and the references of previous reviews, including the IARC monograph on talc, were checked to ensure all available studies were identified. The authors of selected papers were contacted if more complete information was needed.

The eligibility criteria included having cohort with occupational talc exposure and having data (SMR, SIR, or PMR) of lung cancer. Subject to our language ability, only articles written in English or Chinese were considered. Case-control studies were excluded because the odds ratios collected from case-control studies cannot be statistically summarized with the results from talc-exposed cohorts, which are SMRs, SIRs, or PMRs. For systematic quality assessment of the included articles, we applied the Newcastle-Ottawa Scale (NOS) proposed by Wells et al. [21]. The NOS contains an overall nine stars covering three main quality dimensions, including selection of the study population, comparability among the groups, and outcome measures for cohort studies. We used the overall scores to categorize included studies into high (8-9 stars), medium (6-7 stars), and low quality (1–5 stars).

### 2.2. Statistical Analysis

The current meta-analysis was conducted based on procedure described by Sutton [22], in which pooled summary standardized mortality ratios (SMRs) were calculated to identify the risk of lung cancer-related death associated with occupational talc exposure. If a study did not present 95% CIs, Fisher's exact CIs were obtained based on the approximation method described by Armitage et al. [23] using the software package OpenEpi [24]. We used Cochran's* Q* test and* I*-squared to assess heterogeneity in the SMRs reported by the included studies. Given the heterogeneity among included studies in various countries, industries, and coexposures, a random effect approach was adopted as the default analysis, and subgroup analysis was performed by grouping variables to obtain potential explanations. Publication bias was assessed graphically using funnel plots and was also evaluated using Egger's test [25]. Subgroups were further stratified for analysis according to potential confounding variables including asbestos contamination, industry type, and geographic factor. Healthy worker effect was also assessed using the SMRs of all-cause mortality. A sequential exclusion of each study was performed to demonstrate the influence on the meta-SMR by individual studies.

The term asbestiform referred to silicate minerals arranged in poly-filamentous bundles composed of extremely flexible fibers with relatively small diameters and long lengths [26]. Based on the description of talc exposure in the selected articles, we classified the talc exposure as “containing asbestiform fiber” if the talc was fibrous, asbestiform, or interlaced with asbestos. Otherwise, the talc exposure was considered to be “nonasbestiform.”

All statistical analyses were conducted using the Meta and Metafor package in R 3.2.2 software [27]. The significance level was set at 5%.

## 3. Results

**Figure 1:**
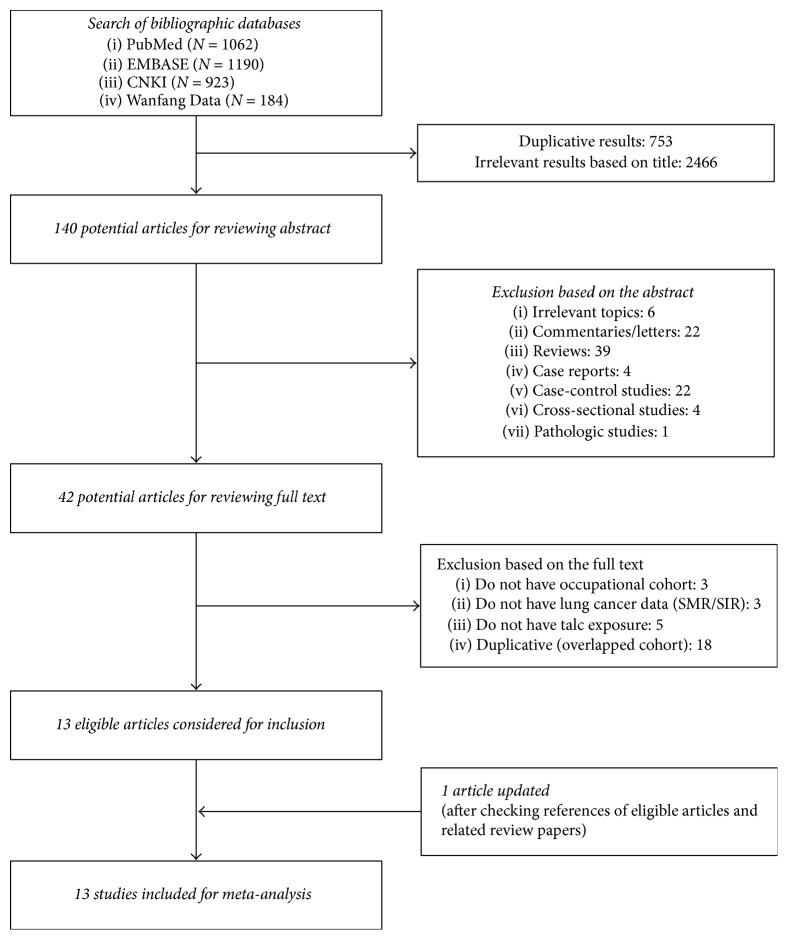
Flowchart of the study selection for the meta-analysis based on prespecified inclusion and exclusion criteria.

The literature search returned 3360 results (PubMed: 1062, EMBASE: 1190, CNKI: 923, and Wanfang Data: 184). Based on a prospectively designed protocol, we identified 140 articles with titles that were relevant to our study purpose. Among them, 42 articles fulfilled our criteria, and their full papers were retrieved. According to the prespecified criteria, data on lung cancer mortality measured by SMR, SIR, or PMR were extracted from 14 cohorts in 13 publications, for one of the included publications contained two separate cohorts.  Figure 1 shows the flowchart used for study selection based on the eligibility and exclusion criteria.

### 3.1. Characteristics of the Studies

Fourteen observational cohort studies (13 publications) were selected for meta-analysis. The characteristics of the included cohorts are summarized in Table 1. The studies that were eligible for inclusion in the meta-analysis included seven cohorts from talc producing companies or talc mines and seven cohorts from user industries. Among them, seven cohorts were exposed to talc without asbestiform fiber contamination. Smoking data were collected in six of the included cohorts (see Table 1). All studies had documented occupational exposures other than talc. The included cohorts had a total population of 95,711. There were 1,766 cases of lung cancer mortality, while expected mortality was 1,302. Lung cancer-related mortality, all-cause mortality, and the NOS quality assessment for each cohort are summarized in Table 2.

### 3.2. Quantitative Synthesis

**Figure 2:**
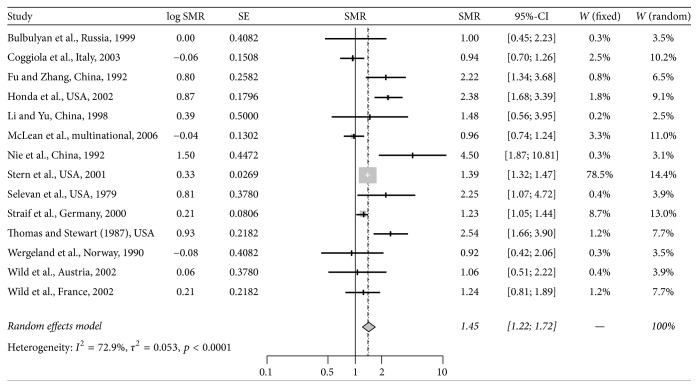
Forest plot of summary SMR for lung cancer pooling all available studies. The forest plot demonstrates that the summary SMR for lung cancer is elevated in subjects exposed to talc powders. LogSMR: estimated log standardized mortality ratio; SE: standard error of LogSMR; SMR: standardized mortality ratio; CI: confidence interval;* W* (fixed): weights from fixed effect;* W* (random): weights from random effect. SMR values > 1.0 indicate that talc exposure is associated with increased mortality risk. Gray squares represent the point estimate of the SMR and have areas proportional to study size. Lines represent 95% confidence intervals. The diamond shows the summary statistic. The overall heterogeneity statistic is shown.

**Figure 3:**
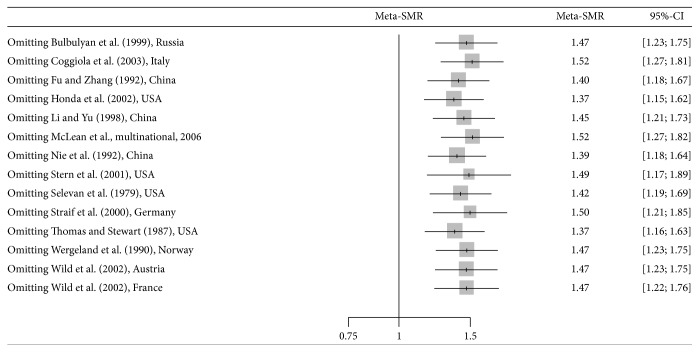
Forest plot of influence of excluding each individual cohort. Gray squares represent the estimate of the meta-SMR and have areas proportional to the pooled study size. Lines represent 95% confidence intervals (random-effects model).

The estimated average SMR for lung cancer was 1.45 (95% CI: 1.22–1.72, *p* < 0.0001) among the workers exposed to talc.  Figure 2 shows the forest plot corresponding to the summary lung cancer SMR calculated in the meta-analysis. The SMRs for the eligible cohorts ranged from 0.92 in a Norwegian cohort [17] to 4.50 in a Chinese cohort [12]. The funnel plot and Egger's test results showed no evidence of publication bias (the* p* value for Egger's test is 0.65). Influence of excluding each individual cohort was summarized in Figure 3 as a forest plot.

### 3.3. Subgroup Analysis for Asbestos Contamination

To prevent confounding from the contamination of asbestiform fibers on lung cancer SMR, subgroup analysis was also performed. In the subgroup where the cohorts were exposed to talc without asbestiform fibers, the meta-SMR was 1.50 (95% CI: 1.02, 2.22; *p* = 0.0391). For the subgroup of cohorts that were exposed to talc contaminated with asbestiform fibers, the meta-SMR was 1.45 (95% CI: 1.18, 1.78; *p* = 0.0004). The test for between-subgroups differences was positive among these three subgroups (Cochran's *Q* = 0.03, df = 1, and *p* value = 0.8680). Our subgroup analysis showed no significant difference in meta-SMR of lung cancer between workers exposed to talc with and without asbestiform fiber contamination.

### 3.4. Subgroup Analysis for Industry Types

To explore the heterogeneity of the study cohorts among different industries, we grouped the included studies into talc-producing industry and user industries (printing industry, rubber industry, pulp and paper industry, cement industry, pottery industry, etc.). The meta-SMR of the talc-producing industry was 1.47 (95% CI: 1.02, 2.10; *p* = 0.0372). The meta-SMR of the talc user industries was 1.42 (95% CI: 1.14, 1.76; *p* = 0.0016). There was no significant difference of the SMR between the talc-producing industry and the user industries.

### 3.5. Subgroup Analysis for Geography

For the geographic dispersion, we grouped the included studies into three regions: Europe, North America, and Asia. The multinational study of McLean et al. [11] was excluded for this analysis. The meta-SMR of the cohorts in Europe was 1.16 (95% CI: 1.02, 1.31; *p* = 0.0281). The meta-SMR of the cohorts in North America was 2.01 (95% CI: 1.34, 3.00; *p* = 0.0007). The meta-SMR of the cohorts in Asia was 1.98 (95% CI: 1.11, 3.50; *p* = 0.0198). The test for between-subgroups differences (random effects model) showed statistical significance (Cochran's *Q* = 9.15, df = 2, *p* = 0.0103).

The results of subgroup analyses were summarized in Table 3, with stratification of cohort studies by subgroups, including type of talc (nonasbestiform talc, talc with asbestiform fiber), industry (talc user industry, talc-producing industry), gender, geography (Asia, Europe, and North America), duration of follow-up (<20 years, 20–40 years, and ≥40 years), publication language (English, Chinese), and study quality.

## 4. Discussion

In the present meta-analysis, we evaluated a wide range of epidemiologic studies related to the topic and found a statistically significant association between occupational talc exposure and lung cancer death. Our study further demonstrated that the increased mortality from lung cancer was observed in cohorts exposed to nonasbestiform talc as well as in those exposed to talc containing asbestiform fibers.

### 4.1. Evidence Updates

In the review of talc by the IARC, talc containing asbestos was classified as “carcinogenic to humans” (group 1), and inhaled talc without asbestos was classified as “not classifiable as to carcinogenicity in humans” (group 3) [3]. A meta-analysis conducted by Wild searched PubMed for articles of talc and cancer before 2004, pooling 42 cases of lung cancer death, and also found no excess lung cancer mortality for the populations of talc millers (meta-SMR = 0.92; 95% CI: 0.67–1.25; fixed effect model), who was exposed to high levels of talc but without any other potential carcinogen [28].

There are three main points between our meta-analysis and the previous one. First, Wild included cohorts from only talc millers (42 cases of lung cancer death). Instead, we included talc-exposing cohorts from all available literatures (1,766 cases of lung cancer death) and conducted further subgroup analysis for several potential confounding factors. We did not classify these cohorts into subgroups before analyzing them collectively, for a priori groupings might have missed other confounding factors or been too small to have adequate statistical power. Second, we included both literatures published in English and Chinese, for China is the largest talc-producing country and many Chinese cohort studies were not translated into English. Third, our meta-analysis searched for literatures through 2016, and the latest publication included in our meta-analysis was in 2006, including the IARC multinational cohort study in 2006 of pulp and paper workers conducted by McLean et al. [11], one of the largest available researches regarding the carcinogenic effect of talc. Supplement Table  1 shows the number of studies and summary estimates from the previous meta-analysis that have been published to compare the current meta-analysis (in Supplementary Material available online at https://doi.org/10.1155/2017/1270608).

### 4.2. Asbestos Contamination

The carcinogenic effect of talc might be confounded by other occupational exposures, such as asbestiform fibers [3, 28]. As previous study stated, the term asbestiform referred to silicate minerals arranged in poly-filamentous bundles composed of extremely flexible fibers with relatively small diameters and long lengths [26]. To examine the effect of this potential confounding factor, we classified the included cohorts into two subgroups, asbestiform subgroup and nonasbestiform subgroup, based on whether workers were exposed to talc with asbestiform fiber contamination, and conducted a sensitivity analysis of the subgroups. The cohorts that were exposed to nonasbestiform talc had a meta-SMR of 1.50 for lung cancer (95% CI: 1.02–2.22), while the cohorts that were exposed to asbestiform fiber-contaminated talc had a meta-SMR of 1.45 for lung cancer (95% CI: 1.18–1.78). There is no significant difference between these two subgroups. As the subgroup analysis showed consistent results, it is more informative to interpret with the overall summary risk [29]. Along with low survival rate of lung cancer, these findings based on mortality data suggest that talc exposure is associated with an increased lung cancer risk, regardless of the presence of asbestiform fibers.

### 4.3. Healthy Worker Effect

Another possible confounding factor is the general condition of the evaluated workers. In the Norwegian study reported by Wergeland et al. and the Austrian study reported by Wild, the authors reported significantly decreased all-cause mortality risks, of which the SMRs were 0.76 (95% CI: 0.62–0.90, SMR of all-site malignancy) and 0.75 (95% CI: 0.58–0.95), respectively [17, 18]. The decreased overall mortality risks indicated that there might be a healthy-worker effect in these studies. These facts might partially explain that previous meta-analysis of talc and cancer tended to yield negative results. On the contrary, the Chinese study reported by Fu and Zhang, the American study reported by Honda et al., and the Chinese study reported by Nie et al. all showed significantly increased all-cause mortality risks, of which the SMRs were 1.27 (95% CI: 1.07–1.49), 1.31 (95% CI: 1.14–1.50), and 1.78 (95% CI: 1.22–2.51), respectively [8, 9, 12]. The general inferior condition of the subjects might therefore reflect the real occupational risk in health. These differences may have resulted from the varying protection measures adopted by the workers and the diverse levels of social economic status of the workers in different countries.

### 4.4. Geographic Dispersion

The different locations used to produce talc may have influenced the associated risk of cancer. Another confounding factor is the geographic dispersion among the studies. For example, Fortunato and Rushton conducted a meta-analysis on the association between asbestos exposure and stomach cancer [30] that grouped cohorts by geographic area into three subgroups: Europe, North America, and Asia. We conducted a subgroup analysis in the current study based on the same geographic groupings, excluding the multinational cohort of McLean et al. [11]. The test for between-subgroups differences was positive among these three subgroups (Cochran's *Q* = 9.15, df = 2, and *p* value = 0.0103). The meta-SMR of lung cancer was 1.98 (95% CI: 1.11, 3.50; number of cohorts = 4) for the talc-exposed cohorts in Asia, 1.16 (95% CI: 1.02, 1.31; number of cohorts = 5) for the cohorts in Europe, and 2.01 (95% CI: 1.34, 3.00, number of cohorts = 4) for the cohorts in North America. Although the result might be biased by variables such as industry types and coexposures, it still indicates that geographic dispersion may have an influence on the mortality risk of lung cancer among the dust-exposed workers, possibly reflecting the levels of labor protection among different countries.

### 4.5. Other Confounding Factors and Influence of Individual Studies

Except for the abovementioned confounding factors, impacts of publication language and the study quality were also found to be significant in the subgroup analyses (Table 3). All three publications (Fu and Zhang [8], Li and Yu [10], and Nie et al. [12]) written in Chinese were conducted in China, showing higher SMRs of lung cancer and higher all-cause mortality than those published in English. This finding was consistent with the findings of analyzing the geographic factor and might also be explained by the varying levels of social economic status and protection measures for workers in different countries. Studies with medium level of NOS score (6-7 stars) reported higher lung cancer mortality than those with high level of NOS score (8-9 stars); however, two of three studies with medium level of NOS score were published in Chinese (Fu and Zhang [8] and Nie et al. [12]). Epidemiologic studies in Chinese scientific journal tend to have shorter paragraphs, which might possibly lead to a lower NOS score due to less detailed description of study methodologies, causing an association between publication language and study quality.

It should be noted that the subjects were coexposed to other hazardous materials in the workplace. To illustrate, the Russian study reported by Bulbulyan et al. collected data from 3,473 female employees working in two printing plants [6]. These workers were coexposed to paper dust, aromatic hydrocarbons, carbon black, and lead, all of which might be possible confounding factors. Similarly, the German study reported by Straif et al. collected data from 8,933 male employees from five rubber plants [15] and reported coexposure to asbestos, nitrosamines, and carbon black.

Some of the studies reported possible misclassification bias and data loss. The Chinese study reported by Zhang et al. collected data from 1,624 rubber workers and reported SMRs for specific causes of death [31]. However, among all the rubber workers included, only the workers engaged in the production of tires and inner tubes were exposed to talc (with asbestiform fibers), and there might be a misclassification bias. Therefore, we replaced the study of Zhang et al. by a newer study of the same cohort conducted by Li and Yu [10] and obtained the subgroup data needed for the meta-analysis. The Norwegian study reported by Langseth and Andersen collected data from 4,247 female workers working in 10 paper mills [32]. The study provided no quantitative assessment of exposure to talc, asbestos, or other compounds. Additionally, although job titles and work histories were obtained, the administrative staff, who usually had no exposure to talc, were not removed from the data analysis. Without considering job types, the risk of lung cancer might have been underestimated. Therefore, we replaced the study of Langseth et al. by the multinational study of McLean et al. [11], which also included this Norwegian cohort and had the subgroup data of workers exposed to talc. The Italian study reported by Coggiola el al. collected data from 1,795 male employees working in an Italian talc mine [7]. The study reported a 7% data missing rate, which might have led to an underestimation of cancer risk. Another source of potential bias in that study is that the job type “millers” might have included some office administrators, indicating that the total cohort SMR may have been underestimated due to misclassification.

### 4.6. Carcinogenic Mechanism of Talc

Currently, the carcinogenic mechanism of talc is suspected to be chronic inflammation [33]. Davies et al. examined the effects of different types of talc on mouse peritoneal macrophages* in vitro*, and the results showed that talc is cytotoxic to macrophages and may be able to induce fibrosis and chronic inflammation in animals [34]. In an animal study evaluating the carcinogenic effects of inhaled talc on both mice and rats, the lungs of the talc-treated groups showed chronic granulomatous inflammation, alveolar epithelial hyperplasia, squamous metaplasia, squamous cysts, and interstitial fibrosis [33]. Exposure to talc was also shown to have an association with chronic inflammation and the accumulation of macrophages, resulting in a potential carcinogenic effect.

### 4.7. Limitations

There are some limitations in the present study. On the one hand, smoking data were not available for all the cohorts. Although smoking is a risk factor for various cancers and should be evaluated, the included studies that collected smoking data all reported that smoking posed no significant confounding effect on mortality risk for the workers compared to the reference population. However, the incomplete smoking data for the cohorts prevented further stratification of the meta-analysis. On the other hand, the gender effect could not be analyzed in the present study because most of the workers exposed to talc were male. Lastly, the lack of quantitative data of talc exposure hindered this study from presenting a positive dose-response relationship. In brief, the association between occupational talc exposure and risk of lung cancer should be further investigated. We suggest future epidemiologic studies of occupational talc exposure that include the quantitative data of exposure and information on smoking and enroll a greater number of females.

## 5. Conclusions

In conclusion, the present study supports a positive association between talc exposure and lung cancer, regardless of whether such exposure is talc with or without asbestos. The safety of workers with occupational talc exposure should be carefully reevaluated.

## Supplementary Material

Supplement Table 1. The table shows the number of studies and summary estimates from the previous meta-analysis by Wild that have been published to compare the current meta-analysis.
Appendix 1: R Script. The R script shows the process of statistical analysis using the Meta and Metafor package in R 3.2.2 software.
Appendix 2: EndNote File. The EndNote file contains the original results of literature search, including the process of filtering and selection of potential articles.

## Figures and Tables

**Table 1:** Summary characteristics of talc-exposed cohorts eligible for meta-analysis.

Reference/location	Cohort definition	Exposure assessment	Talc exposure	Other exposures	Smoking
Bulbulyan et al., Russia, 1999 [6]	3473 female employees of two printing plants employed >2 years, 1978–93, followed up for cancer incidence from 1979 to 1993	Area air sampling data plus job description data; job type: compositors, press operators, bookbinders, and other	Talc with asbestos contamination	Paper dust, benzene, aromatic hydrocarbons, carbon black, lead	Unknown

Coggiola et al., Italy, 2003 [7]	1795 male employees from Italian talc mine employed >1 year, 1946–1995, followed up from 1946 to 1995	Job histories from plant records; job type: miner versus miller; duration of exposure and time since first exposure (years)	Talc without asbestiform fibers; >200 mppcf in 1950, <5 mppcf in 1965	Quartz, radon (miners)	44%–47% smokers compared to 34% in Italian population (1994)

Fu and Zhang, China, 1992 [8]	1357 male workers in Haichen talc mines employed >1 year in January 1974, followed up through 1988	Job histories from factory records; job type: miner versus miller; medical exam records in local hospitals; duration of exposure and time since first exposure (years)	Talc without asbestiform fibers	Radon (miners)	64% smokers

Honda et al., USA, 2002 [9]	782 male employees from a New York talc mining and milling facility employed >1 day, 1948–1989, followed up from 1950 to 1989	Cumulative respirable dust exposure estimation for individual subjects from a job-exposure matrix consisting of estimates of respirable dust concentrations for all work area and calendar year combinations	Talc with asbestos contamination; 0.1–1.7 mg/m^3^	Asbestos, nonasbestiform amphibole, taconite	Unknown

Li and Yu, China, 1998 [10]	934 male and 664 female workers of a Shanghai rubber factory who entered a screening program for heart disease in 1972, followed up from 1973 to 1995	Based on information on work history obtained from the records of a screening program for heart disease	Talc with asbestos contamination was exposed to the workers engaged in the production of tires and inner tubes	Nitrosamine, multiple solvents	63% for male workers and 9% for female workers compared to 46% for men and 5% for women in general population

McLean et al., multinational, 2006 [11]	60,468 workers employed > 1 year in the pulp and paper industry in 11 countries, 1920–1996; followed up through 1996	Exposure was estimated at the department level based on assessments of an international panel of industrial hygiene experts through detailed company questionnaires	Talc with asbestos contamination, categorized into ever-exposure and ever-high-exposure levels	Paper dust, asbestos, multiple volatile, and nonvolatile organochlorine compounds	Unknown

Nie et al., China, 1992 [12]	8654 male and 3564 female pottery workers (412 with talc exposure) employed >1 year, 1972–1974, followed up through 1989	Area air sampling data plus work histories; minerals analyzed by phase contrast microscopy	Talc without asbestiform fibers; 1–18% of total dust	Silica	Unknown

Stern et al., USA, 2001 [13]	12873 males of the Operative Plasterers' and Cement Masons' International Association, 1972–1996; followed up through 1996	Potential worker exposures were based on a representative sample of 4,500 U.S. industrial facilities conducted by the USA National Occupational Exposure Survey (NOES) during 1981–1983	Talc with asbestos contamination	Cement dust, 1,1,1- trichloroethylene, quartz, sand, mica, silica, attapulgite, asphalt, brick clay, carbon tetrachloride, dioxane, tetrachloroethylene, fiberglass	Unknown

Selevan et al., USA, 1979 [14]	388 male talc workers from 5 talc producing companies in Vermont employed >1 year, 1940–1969, followed up from 1940 to 1975	Based on historical data that demonstrated past exposure levels far exceeded the standard for nonfibrous talc of 20 mppcf	Talc without asbestiform fibers; commonly >20 mppcf	Quartz (<0.25%)	Unknown

Straif et al., Germany, 2000 [15]	8933 male employees from 5 German rubber plants employed >1 year retired or active in 1981 followed up from 1981 to 1991	Work histories reconstructed from cost center codes plus semiquantitative cumulative exposure	Talc with asbestos contamination	Asbestos, nitrosamines, carbonblack	Unknown

Thomas and Stewart, USA, 1987 [16]	2055 white male workers from American pottery factory employed >1 year, 1939–66; mortality follow-up through 1981	Exposure to silica and talc assessed qualitatively by job title and department by industrial hygienist	Nonfibrous talc (a subgroup of workers exposed only to silica and nonfibrous talc)	Quartz	Unknown

Wergeland et al., Norway, 1990 [17]	389 male employees from a Norwegian talc mill employed >2 years, 1935–1972, followed up from 1953 to 1987	Subjective assessment of exposure by experienced employees; workers classified based on low, medium, high, and unknown exposure by total duration of employment in jobs	Talc without asbestiform fibers. For miners: 0.94–97.35 mg/m^3^ peaked at 319 mg/m^3^ 0.2–0.9 f/ml. For millers: 1.4–54.1 mg/m^3^ peaked at 109 mg/m^3^ 0.2–0.9 f/ml	Radon (miners)	76% smokers (miners)

Wild et al., Austria, 2002 [18]	542 male workers of an Austrian talc producing company employed >1 year, 1972–1995, followed up from 1972 to 1995	Semiquantitative, site-specific job-exposure matrix based on personal dust measurements and descriptions from employees	Talc without asbestiform fibers; >30 mg/m^3^ before 1960, 5–30 mg/m^3^ until 1980, <5 mg/m^3^ thereafter	Quartz (<3%)	42% smokers

Wild et al., France, 2002 [18]	945 male employees from a French talc mill employed >1 year, 1945–1994, followed up from 1968 to 1995	Semiquantitative, site-specific job-exposure matrix based on personal dust measurements and descriptions from employees	Talc without asbestiform fibers; >30 mg/m^3^ before 1970s, 5–30 mg/m^3^ until 1990, <5 mg/m^3^ thereafter	Quartz (<3%)	59% smokers compared to 39% French population (1986)

**Table 2:** Lung cancer risk of talc-exposed cohorts eligible for meta-analysis.

Reference/location	Newcastle-Ottawa Scale (NOS) criteria^a^				Cases	Type of RR	RR	95% CI	Mortality, all causes
	Selection	Comparability	Outcome	Overall quality (total score)^e^					
Bulbulyan et al., Russia, 1999	(+) (+) (+) ( )	(+) (+)	(+) (+) (+)	High (8)	6	SMR	1.00	0.37–2.18	0.98
Coggiola et al., Italy, 2003	(+) (+) (+) ( )	(+) (+)	(+) (+) (+)	High (8)	44	SMR	0.94	0.68–1.26	1.20
Fu and Zhang, China, 1992	(+) ( ) (+) ( )	(+) (+)	(+) (+) (+)	Medium (7)	15	SMR	2.22	1.24–3.66	1.27
Honda et al., USA, 2002	(+) (+) (+) ( )	(+) (+)	(+) (+) (+)	High (8)	31	SMR	2.38	1.57–3.29	1.31
Li and Yu, China, 1998	(+) (+) (+) ( )	(+) (+)	(+) (+) (+)	High (8)	4^b^	SMR	1.48	0.40–3.79	1.16
McLean et al., multinational, 2006	(+) (+) (+) ( )	(+) (+)	(+) (+) (+)	High (8)	59^c^	SMR	0.96	0.73–1.24	0.88
Nie et al., China, 1992	(+) (+) (+) ( )	(+) (+)	(+) (+) ( )	Medium (7)	5	SMR	4.50	1.46–10.50	1.78
Stern et al., USA, 2001	(+) (+) (+) ( )	(+) (+)	(+) (+) (+)	High (8)	1386	PMR	1.39	1.32–1.47	1.00
Selevan et al., USA, 1979	(+) (+) ( ) ( )	(+) (+)	(+) (+) (+)	Medium (7)	7	SMR	2.25	0.09–3.69	1.22
Straif et al., Germany, 2000	(+) (+) (+) (+)	(+) (+)	(+) (+) (+)	High (9)	154	SMR	1.23	1.04–1.44	1.03
Thomas and Stewart, USA, 1987	(+) (+) (+) ( )	(+) (+)	(+) (+) (+)	High (8)	21	SMR	2.54	1.57–3.88	0.9^d^
Wergeland et al., Norway, 1990	(+) (+) (+) ( )	(+) (+)	(+) (+) (+)	High (8)	6	SIR	0.92	0.34–2.01	0.75
Wild et al., Austria, 2002	(+) (+) (+) ( )	(+) (+)	(+) (+) (+)	High (8)	7	SMR	1.06	0.43–2.19	0.75
Wild et al., France, 2002	(+) (+) (+) ( )	(+) (+)	(+) (+) (+)	High (8)	21	SMR	1.24	0.76–1.89	0.93

^a^The items of Newcastle-Ottawa Scale (NOS) for cohort studies are categorized into selection, comparability, and outcome. The list of items: representativeness of the exposed cohort (Selection-1), selection of the nonexposed cohort (Selection-2), ascertainment of exposure (Selection-3), demonstration that outcome of interest was not present at start of study (Selection-4), comparability of cohorts on the basis of the design or analysis (Comparability-1), assessment of outcome (Outcome-1), long enough follow-up for outcomes to occur (Outcome-2), and adequacy of follow-up of cohorts (Outcome-3). ^b^The SMR (observed cases = 3, expected cases = 2.4) was calculated based on the workers engaged in the production of tires and inner tubes, who were categorized as the talc-exposed group. ^c^The SMR (observed cases = 24, expected cases = 18.69) was calculated based on the workers ever highly exposed to talc. For the workers ever exposed to talc, the number of observed cases was 104, and the number of expected cases was 113.16. These supplementary data were obtained by email contacts to the authors [11]. ^d^The SMR of lung cancer was calculated based on the workers exposed to nonfibrous talc, but the SMR of all-cause mortality was only available for the total cohort. ^e^The overall scores of NOS were categorized into three levels: high (8-9 stars), medium (6-7 stars), and low quality (1–5 stars).

**Table 3:** Stratification of cohort studies by subgroups.

	*n* ^a^	Meta-SMR	95% CI	*p* value^b^
Type of talc				
Nonasbestiform talc	7	1.51	1.02–2.22	0.87
Talc with asbestiform fiber	7	1.45	1.18–1.78	
Industry				
Talc user industry	7	1.41	1.14–1.76	0.87
Talc-producing industry	7	1.47	1.02–2.11	
Gender				
Female	3	1.71	0.71–4.12	0.66
Male	12	1.39	1.16–1.67	
Geography				
Asia	4	1.98	1.11–3.51	0.01
Europe	5	1.16	1.02–1.31	
North America	4	2.01	1.34–3.00	
Duration of follow-up				
<20 years	4	1.76	1.03–3.00	0.72
20–40 years	7	1.51	1.18–1.92	
≥40 years	3	1.28	0.75–2.19	
Publication language				
English	11	1.34	1.13–1.60	0.04
Chinese	3	2.45	1.44–4.16	
Study quality (NOS score)				
High (8-9)	11	1.32	1.74–3.71	<0.01
Medium (6-7)	3	2.54	1.11–1.56	
Low (1–5)	0			

^a^Number of cohorts included.  ^b^Test for between-subgroup differences (random effects model).

## Abbreviations

CI: Confidence interval
CNKI: China National Knowledge Infrastructure
EMP: Elongated mineral particle
FDA: Food and Drug Administration
IARC: International Agency for Research on Cancer
mppcf: Million particles per cubic foot
NIOSH: National Institute for Occupational Safety and Health
NOS: Newcastle-Ottawa Scale
NTP: National Toxicology Program
PMR: Proportionate mortality ratio
SIR: Standardized incidence ratio
SMR: Standardized mortality ratio
WHO: World Health Organization.

## Additional Points

*Strengths and Limitations of the Study*. (i) This study shows an association between occupational talc exposure and mortality of lung cancer via meta-analysis. (ii) This study includes epidemiological studies from China, the largest talc producing country. (iii) The main limitation of this study is lack of dose-response evidence.
