# Supplementary material for: Occupational Exposure to Talc Increases the Risk of Lung Cancer: A Meta-Analysis of Occupational Cohort Studies
Source: Can Respir J. 2017 Aug 31;2017:1270608. doi: 10.1155/2017/1270608 (PMC5610800; doi:10.1155/2017/1270608)
Supplement: Supplementary file 1 — Supplement Table 1. The table shows the number of studies and summary estimates from the previous meta-analysis by Wild that have been published to compare the current meta-analysis. Appendix 1: R Script. The R script shows the process of statistical analysis using the Meta and Metafor package in R 3.2.2 software. Appendix 2: EndNote File. The EndNote file contains the original results of literature search, including the process of filtering and selection of potential articles. [file 1270608.f1.pdf]

	Current study	Wild et al. 2006
**Cohort included**		
Bulbulyan, Russia, 1999	Included [a]	Not included
Coggiola, Italy, 2003	Included [b]	Included [c]
Fu, China, 1992	Included [b]	Not included
Honda, USA, 2002	Included [a]	Not included
Li, China, 1998	Included [a]	Not included
Mclean, multinational, 2006	Included [a]	Not included
Nie, China, 1992	Included [b]	Not included
Stern, USA, 2001	Included [a]	Not included
Selevan, USA, 1979	Included [b]	Included [c]
Straif, Germany, 2000	Included [a]	Not included
Thomas, USA, 1987	Included [b]	Not included
Wergeland, Norway, 1990	Included [b]	Included [c]
Wild, Austria, 2002	Included [b]	Included [c]
Wild, France, 2002	Included [b]	Included [c]
Summary estimates	Non-asbestiform talc: meta-SMR =1.50 95% CI = 1.02-2.22 (Random effect)	Non-asbestiform talc: meta-SMR = 0.92 95% CI = 0.67-1.25 (Fixed effect)

a. Talc with asbestiform fiber / asbestos; calculation of meta-SMR was separated from cohorts exposed to non-asbestiform talc

b. Non-asbestiform talc

c. Only talc millers were included

Description: The R script for the process of statistical analysis using the Meta and Metafor package in R 3.2.2 software. Additional information including the raw data are provided within the code.

Downloadable link: https://goo.gl/wfDNzt

```
# Talc Exposure and Risk of lung Cancer: A Meta-Analysis of Occupational Cohort
Studies

# set-up:
rm(list=ls(all=TRUE))
ls()

# Load required packages: meta, metafor
if(require(meta)==FALSE){install.packages("meta");require(meta)}
if(require(metafor)==FALSE){install.packages("metafor");require(metafor)}
if(require(gsheet)==FALSE){install.packages("gsheet");require(gsheet)}  # for reading
online spreadsheet

# continuous data:
meta04lung <-
gsheet2tbl('https://docs.google.com/spreadsheets/d/1BYMCu3gKjjY5fYXPfIlxdufU8K0JSX
WB1-EIVym_1j8/edit#gid=1782588271')
summary(meta04lung)
meta04lung$logsmr = log(meta04lung$obs / meta04lung$exp)
meta04lung$selogsmr = 1 / sqrt(meta04lung$obs)
meta04lung.gen = metagen(logsmr, selogsmr, study, sm="HR",data=meta04lung,
comb.fixed = F)
meta04lung.gen
forest(meta04lung.gen, smlab = "SMR", leftlabs = c("Study", "logSMR", "SE"), rightlabs
= c("SMR", "95%-CI", "Weights"))
funnel(meta04lung.gen)
# Egger's test for publication bias
metabias(meta04lung.gen, method.bias = "linreg", plotit = T)

# Sensitivity analysis: asbestos contamination
meta04lung.byvar = metagen(logsmr, selogsmr, study, sm="HR",data=meta04lung, byvar
= contamination, comb.fixed = F)
meta04lung.byvar
forest(meta04lung.byvar, smlab = "SMR", leftlabs = c("Study", "logSMR", "SE"),
rightlabs = c("SMR", "95%-CI", "Weights"))
# contamination = 0
```

```
# Sensitivity analysis: talc-producing vs. user industries
meta04lung$talcmine = c(0,1,1,1,0,0,0,0,1,0,0,1,1,1) # 1 = talc-producing, 0 = user
industries
meta04lung.byvar = metagen(logsmr, selogsmr, study, sm="HR",data=meta04lung, byvar
= talcmine, comb.fixed = F)
meta04lung.byvar
forest(meta04lung.byvar, smlab = "SMR", leftlabs = c("Study", "logSMR", "SE"),
rightlabs = c("SMR", "95%-CI", "Weights"))
# user industries
metagen(logsmr, selogsmr, study, sm="HR",data=subset(meta04lung, talcmine==0),
comb.fixed = F)
# talc-producing industry
metagen(logsmr, selogsmr, study, sm="HR",data=subset(meta04lung, talcmine==1),
comb.fixed = F)

# Sensitivity analysis: geography
meta04lung$geography01 = c(1,2,1,3,1,4,1,3,3,2,3,2,2,2) # 1=Asia, 2=Europe, 3=North
America, 4=multi-national
meta04lung.byvar = metagen(logsmr, selogsmr, study, sm="HR",data=meta04lung[-6,],
byvar = geography01, comb.fixed = F)
meta04lung.byvar
forest(meta04lung.byvar, smlab = "SMR", leftlabs = c("Study", "logSMR", "SE"),
rightlabs = c("SMR", "95%-CI", "Weights"))
# Asia (China + Russia)
metagen(logsmr, selogsmr, study, sm="HR",data=subset(meta04lung[-6,],
geography01==1), comb.fixed = F)
  # Russian vs. Chinese studies
  metagen(logsmr, selogsmr, study, sm="HR",data=subset(meta04lung[-6,],
geography01==1), byvar = language, comb.fixed = F)
# Europe
metagen(logsmr, selogsmr, study, sm="HR",data=subset(meta04lung[-6,],
geography01==2), comb.fixed = F)
# North America
metagen(logsmr, selogsmr, study, sm="HR",data=subset(meta04lung[-6,],
geography01==3), comb.fixed = F)

# Sensitivity analysis: duration of follow-up
meta04lung$duration = cut(meta04lung$duration_followed, c(0, 20, 40, 100), right = F,
labels = c("<20 years", "20-40 years", ">=40 years"))
summary(meta04lung$duration)
meta04lung.byvar = metagen(logsmr, selogsmr, study, sm="HR",data=meta04lung, byvar
= duration, comb.fixed = F)
meta04lung.byvar
forest(meta04lung.byvar, smlab = "SMR", leftlabs = c("Study", "logSMR", "SE"),
```

```
metagen(logsmr, selogsmr, study, sm="HR",data=subset(meta04lung, duration_followed >= 20 & duration_followed < 40), comb.fixed = F)
# >=40 years
metagen(logsmr, selogsmr, study, sm="HR",data=subset(meta04lung, duration_followed >= 40), comb.fixed = F)

# Sensitivity analysis: language
summary(meta04lung$language)
meta04lung.byvar = metagen(logsmr, selogsmr, study, sm="HR",data=meta04lung, byvar = language, comb.fixed = F)
meta04lung.byvar
forest(meta04lung.byvar, smlab = "SMR", leftlabs = c("Study", "logSMR", "SE"), rightlabs = c("SMR", "95%-CI", "Weights"))
# English
metagen(logsmr, selogsmr, study, sm="HR",data=subset(meta04lung, language == "English"), comb.fixed = F)
# Chinese
metagen(logsmr, selogsmr, study, sm="HR",data=subset(meta04lung, language == "Chinese"), comb.fixed = F)


# Sensitivity analysis: study quality (NOS score)
meta04lung$quality = cut(meta04lung$NOS_score, c(6, 8, 10), right = F, labels = c("medium", "high"))
summary(meta04lung$quality)
meta04lung.byvar = metagen(logsmr, selogsmr, study, sm="HR",data=meta04lung, byvar = quality, comb.fixed = F)
meta04lung.byvar
forest(meta04lung.byvar, smlab = "SMR", leftlabs = c("Study", "logSMR", "SE"), rightlabs = c("SMR", "95%-CI", "Weights"))
# medium
metagen(logsmr, selogsmr, study, sm="HR",data=subset(meta04lung, quality == "medium"), comb.fixed = F)
# high
metagen(logsmr, selogsmr, study, sm="HR",data=subset(meta04lung, quality == "high"), comb.fixed = F)


# Influence of excluding each individual cohort (leave-one-out method)
meta04lung.gen.inf = metainf(meta04lung.gen, pooled = "random")
meta04lung.gen.inf
forest(meta04lung.gen.inf, overall = F, smlab = "Meta-SMR", leftlabs = c(""), rightlabs = c("Meta-SMR", "95%-CI"))
```

```
# Sensitivity analysis: gender
meta04lung_gender <-
gsheet2tbl('https://docs.google.com/spreadsheets/d/1BYMCu3gKjjY5fYXPfIlxdufU8K0JSX
WB1-EIVym_1j8/edit#gid=29647490')
summary(meta04lung_gender)
meta04lung_gender$logsmr = log(meta04lung_gender$obs / meta04lung_gender$exp)
meta04lung_gender$selogsmr = 1 / sqrt(meta04lung_gender$obs)
meta04lung_gender.byvar = metagen(logsmr, selogsmr, study,
sm="HR",data=meta04lung_gender[-9,], byvar = Gender, comb.fixed = F)
meta04lung_gender.byvar
forest(meta04lung_gender.byvar, smlab = "SMR", leftlabs = c("Study", "logSMR", "SE"),
rightlabs = c("SMR", "95%-CI", "Weights"))
# female
metagen(logsmr, selogsmr, study, sm="HR",data=subset(meta04lung_gender,
Gender=="female"), comb.fixed = F)
# male
metagen(logsmr, selogsmr, study, sm="HR",data=subset(meta04lung_gender,
Gender=="male"), comb.fixed = F)
```

Description: The EndNote file contains the original results of literature search, including the process of filtering and selection.

Downloadable link: https://goo.gl/A5Yojg
